# Modulation of sirtuins during monolayer chondrocyte culture influences cartilage regeneration upon transfer to a 3D culture environment

**DOI:** 10.3389/fbioe.2022.971932

**Published:** 2022-12-06

**Authors:** Hannah K. Heywood, Stephen D. Thorpe, Renos M. Jeropoulos, Paul W. Caton, David A. Lee

**Affiliations:** ^1^ School of Engineering and Materials Science, Queen Mary University of London, London, United Kingdom; ^2^ UCD School of Medicine, UCD Conway Institute of Biomolecular and Biomedical Research, University College Dublin, Dublin, Ireland; ^3^ Trinity Centre for Biomedical Engineering, Trinity College Dublin, Dublin, Ireland; ^4^ Barts and the London School of Medicine and Dentistry, Queen Mary University of London, London, United Kingdom; ^5^ Department of Diabetes, School of Life Course Sciences, King’s College London, London, United Kingdom

**Keywords:** chondrocyte, cartilage tissue engineering, sirtuin, glucose restriction, pellet culture, nicotinamide adenine dinucleotide

## Abstract

This study examined the role of sirtuins in the regenerative potential of articular chondrocytes. Sirtuins (SIRT1-7) play a key role in regulating cartilage homeostasis. By inhibiting pro-inflammatory pathways responsible for cartilage degradation and promoting the expression of key matrix components, sirtuins have the potential to drive a favourable balance between anabolic and catabolic processes critical to regenerative medicine. When subjected to osmolarity and glucose concentrations representative of the *in vivo* niche, freshly isolated bovine chondrocytes exhibited increases in *SIRT1* but not *SIRT3* gene expression. Replicating methods adopted for the *in vitro* monolayer expansion of chondrocytes for cartilage regenerative therapies, we found that *SIRT1* gene expression declined during expansion. Manipulation of sirtuin activity during *in vitro* expansion by supplementation with the SIRT1-specific activator SRT1720, nicotinamide mononucleotide, or the pan-sirtuin inhibitor nicotinamide, significantly influenced cartilage regeneration in subsequent 3D culture. Tissue mass, cellularity and extracellular matrix content were reduced in response to sirtuin inhibition during expansion, whilst sirtuin activation enhanced these measures of cartilage tissue regeneration. Modulation of sirtuin activity during monolayer expansion influenced H3K27me3, a heterochromatin mark with an important role in development and differentiation. Unexpectedly, treatment of primary chondrocytes with sirtuin activators in 3D culture reduced their matrix synthesis. Thus, modulating sirtuin activity during the *in vitro* monolayer expansion phase may represent a distinct opportunity to enhance the outcome of cartilage regenerative medicine techniques.

## 1 Introduction

It is estimated that around 10,000 people in the United Kingdom each year experience cartilage damage that warrants treatment (NICE, 2017). If untreated, such cartilage injuries impair quality of life and increase the risk of osteoarthritis (OA). This has motivated the development of cell-based cartilage repair techniques such as autologous chondrocyte implantation (ACI) and evolving tissue engineering/regenerative medicine approaches (Brittberg et al., 1994; Huang et al., 2016). In many cell-based repair techniques chondrocytes are isolated from a cartilage biopsy and increased in number by maintaining the cells in monolayer culture, such that a much larger cell population can then be re-implanted into the defect to repair the cartilage. However, the capacity of cells to regenerate cartilage upon re-implantation progressively declines during *in vitro* culture due to the process of de-differentiation, with associated loss of expression of cartilage extracellular matrix (ECM) macromolecules such as type II collagen and aggrecan (Dell’Accio et al., 2001; Giovannini et al., 2010; Niemeyer et al., 2012). The development of methods that retain chondrocyte regenerative potential during monolayer culture would provide a major advance for cartilage repair techniques, potentially enhancing clinical outcomes (Saris et al., 2009; Niemeyer et al., 2012).

Sirtuins are a family of seven enzymes (SIRT1-7) which have been established to play a key role in regulating cartilage homeostasis. To date, most studies have focussed on SIRT1 and have revealed that SIRT1 protects against cartilage degradation through multiple mechanisms to delay the onset and progression of both age-associated and instability-linked OA (Matsuzaki et al., 2014). However, transcription and protein levels of SIRT1 decline during ageing and reduced SIRT1 levels correlate with increased severity of OA (Fujita et al., 2011; Li et al., 2016). Importantly, the genetic manipulation of SIRT1 expression has revealed a causal link between SIRT1 deficiency and OA disease progression in animal models (Gabay et al., 2013a; Gabay et al., 2013b). Further studies have demonstrated that SIRT3 and SIRT6 also promote cartilage health (Nagai et al., 2015; Wu et al., 2015; Fu et al., 2016), while a contrasting role was recently revealed for SIRT7 as a negative regulator of cartilage homeostasis, through suppression of the transcriptional activity of pro-chondrogenic SOX9 (Korogi et al., 2018).

The sirtuins are nicotinamide adenine dinucleotide (NAD)-dependent de-acetylases which target histones, transcription factors and enzymes to regulate cell behaviour. SIRT1 de-acetylates the pro-inflammatory nuclear factor-kappa B (NF-κB) transcription factor (Yeung et al., 2004; Lei et al., 2012), inactivating it and thereby inhibiting the pro-inflammatory pathways responsible for cartilage degradation mediated by matrix metalloproteinases (MMPs) and a disintegrin-like and metalloproteinases with thrombospondin motifs (ADAMTS) (Kiernan et al., 2003; Yeung et al., 2004; Matsushita et al., 2013; Moon et al., 2013; Elayyan et al., 2017). SIRT1 also transcriptionally suppresses the senescence marker p16 through epigenetic modification at the p16 promoter (Li and Tollefsbol, 2011) and downregulates pro-apoptotic proteins such as p53 to promote cell survival under stress (Luo et al., 2001; Vaziri et al., 2001; Takayama et al., 2009). Conversely, SIRT1-mediated de-acetylation activates the pro-chondrogenic transcription factor SOX9, promoting expression of key matrix components aggrecan and type II collagen (Dvir-Ginzberg et al., 2008; Fujita et al., 2011; Bar Oz et al., 2016). Thus, sirtuins have the potential to drive a favourable balance between anabolic and catabolic processes that is critical to regenerative medicine. However, the importance of sirtuin activity during monolayer expansion for cell-based repair techniques has not been examined previously.

*In vivo*, chondrocytes experience high osmolarity conditions and low glucose levels compared to other tissues. In articular cartilage the Donnan osmotic effect of negatively charged proteoglycan in the ECM increases osmolarity of the cartilage niche from ∼350 mOsm in the superficial zone to 450 mOsm in the deeper regions, and this is increased further by joint loading (Maroudas and Evans, 1972; Oswald et al., 2008). Culture media has been optimised to replicate the osmotic environment of other tissues with the result that media typically used for *in vitro* chondrocyte culture has a lower osmolarity than these specialised cells experience *in vivo*, at approximately 250–280 mOsm. Conversely, typical culture media contains 10 mM glucose which far exceeds *in vivo* concentrations which can be as low as 1 mM in the deep zone of articular cartilage (Windhaber et al., 2003). The transition from cartilage tissue to monolayer culture is therefore accompanied by a shift from high osmolarity and low glucose to low osmolarity and high glucose. It is well established that glucose restriction promotes sirtuin activity (Lin et al., 2002; Li and Tollefsbol, 2011), and we demonstrated previously that chondrocytes expanded in physiological glucose levels of 1 mM subsequently generated greater tissue mass in pellet culture than 10 mM glucose typical of *in vitro* expansion conditions (Heywood et al., 2014). However, the mechanism remains to be established and the potential role of sirtuins in this phenomenon is not known.

A number of small molecule activators of sirtuins have been reported to ameliorate the decline in sirtuin function in aging and disease. Resveratrol is known to augment sirtuin activity in cartilage and other tissues, although the exact mechanism of action has been debated and it affects many other targets. The activity of sirtuins is regulated by the availability of their common substrate, NAD (Imai and Guarente, 2014). Several NAD precursors have emerged, including nicotinamide riboside (NR) and nicotinamide mononucleotide (NMN) which have been demonstrated to successfully enhance sirtuin activity when supplemented in the diet, or in culture medium of cells *in vitro* (Imai, 2010). In addition, novel potent synthetic activators have been developed, such as the SIRT1-specific activator, SRT1720 (Milne et al., 2007). Therefore, this study tests the hypothesis that activation of sirtuins by supplementation with SRT1720 or NMN during chondrocyte maintenance *in vitro* augments subsequent cartilage regeneration potential. This study demonstrates, for the first time, that treatment with small molecule activators of sirtuin activity during the *in vitro* cell expansion phase may augment the regenerative potential of the expanded chondrocytes. Ultimately, this study aims to enhance the clinical success of cartilage repair procedures such as ACI.

## 2 Materials and methods

### 2.1 Ethics statement

Bovine metacarpophalangeal joints were procured as surplus to other food/agricultural use from an abattoir in the United Kingdom, operated under the relevant regulations on animal welfare. This study does not use tissue from animals maintained for research purposes.

### 2.2 Chondrocyte isolation and seeding

Cartilage was dissected under sterile conditions and chondrocytes were isolated from the tissue by sequential incubation at 37°C in complete Dulbecco’s modified Eagle medium (DMEM) supplemented with 20% (v/v) foetal bovine serum (DMEM + 20% FBS) and 5.7 mg/ml pronase for 1 h followed by incubation in DMEM + 20% FBS with 100 U/ml collagenase overnight, as described previously (Heywood and Lee, 2017). All reagents were from Sigma-Aldrich (Poole, United Kingdom). Cells obtained from different donors were isolated and maintained separately, reflecting cell therapy practices, and cultures from each donor allocated to all treatment groups. Freshly isolated chondrocytes were maintained in suspension culture for short term experiments or seeded either into culture dishes or onto serum coated coverslips, at a density of 2 × 10^4^ cells/cm^2^ and cultured under standard 5% (v/v) CO_2_ conditions in culture media as outlined below.

### 2.3 Chondrocyte expansion and sirtuin modulation

Control culture media consisted of DMEM+10% (v/v) FBS containing 10 mM glucose and supplemented with 2 mM L-glutamine, 18 mM HEPES buffer, 88 U/ml penicillin, and 88 mg/ml streptomycin (all from Sigma-Aldrich). Media containing 1 mM glucose was supplemented with 9 mM galactose to maintain osmotic balance, as described previously (Heywood et al., 2014). Osmolarity of 10 mM glucose base media was increased through the addition of D-mannitol (Sigma-Aldrich). Osmolarity was confirmed using a freezing-point depression osmometer (model 3250, Advanced Instruments, Norwood, MA). For short term studies on the role of osmolarity or glucose, samples of 1 × 10^6^ freshly isolated chondrocytes were incubated in suspension with appropriate culture media for 5 h or 6 h respectively. Cells were passaged at 85%–90% confluence using trypsin-EDTA (Sigma-Aldrich) to recover the cells, with cell number determined using a haemocytometer, before re-seeding. Cell proliferation at intervening time points was monitored by manually counting cells from representative fields of view from each flask, with phase contrast images captured daily using a microscope-mounted camera. Once the cells had reached a total of 2, 4, 10, and 25 population doublings (PDs), cell extracts for gene expression analysis were collected and cells plated onto coverslips were prepared for immunofluorescent staining. 0 PD cells for gene expression analysis were maintained in culture dishes for 24 h following isolation with treatments as indicated. At 4 PDs cells were detached with trypsin-EDTA, washed and resuspended in fresh culture media before preparation of cell pellets as described below.

Modulators of sirtuin activity were 10 mM nicotinamide (NAM), 100 µM nicotinamide mononucleotide (NMN; both Sigma Aldrich) or 100 nM SRT1720 (Merck, Watford, United Kingdom). Solutions of NAM and NMN were prepared in culture media and SRT1720 was prepared as a stock solution at 25 mM in DMSO before dilution in culture media, such that DMSO represented less than 0.001% v/v at the final concentration of 100 nM. The efficacy of NMN to increase chondrocyte NAD levels was confirmed using a commercial fluorometric NAD assay kit (ab176723, Abcam, Cambridge, United Kingdom) after 4 h exposure using the manufacturer’s methods. Chondrocytes supplemented with the nicotinamide phosphoribosyltransferase (NAMPT) inhibitor FK866 (Sigma Aldrich) at 10 nM, which decreases cellular NAD, acted as a negative control (Supplementary Figures S1A,B), whereas the SRT1720 dose was selected from viability dose-response curves (Supplementary Figure S1C) and published efficacy (Milne et al., 2007).

### 2.4 3D chondrogenic pellet culture

Freshly isolated chondrocytes (0 PD) or chondrocytes cultured to 4 PDs were centrifuged at 380 × *g* such that a cell mass formed at the base of the centrifuge tube. Cell pellets consisting of 0.5 × 10^6^ or 1 × 10^6^ chondrocytes were cultured in 1 ml or 2 ml of control media respectively, with the addition of 50 µM ascorbate (Sigma-Aldrich), such that the treatments applied during the preceding monolayer phase were discontinued. Cell pellets were maintained with loose caps at 37°C and 5% CO_2_, with medium replacement every 2–3 days. After culture for up to 28 days, the wet weight of the cell pellets was recorded and the pellets frozen prior to biochemical analysis of ECM constituents. In addition, NMN (100 µM) or SRT1720 (100 nM) were included in the culture media of pellets formed from freshly isolated cells, to examine the effect of treatments on differentiated cells during the 3D culture phase.

### 2.5 Biochemical analysis

Samples for biochemical analysis were digested with papain as described previously (Heywood et al., 2004), prior to quantification of sulphated glycosaminoglycan (sGAG) content using the dimethylmethylene blue assay (Farndale et al., 1986), and estimation of collagen content using the hydroxyproline assay (Kafienah and Sims, 2004). DNA content was assessed with a fluorometric assay using Hoechst 33258 (Sigma-Aldrich) as established by Kim et al. (1988).

#### 2.6 qPCR

Gene expression following expansion for up to 25 PDs was determined by quantitative polymerase chain reaction (qPCR). Total RNA of monolayer cells was extracted using TRIzol reagent (Invitrogen) according to the manufacturer’s instructions. RNA yield and quality was assessed using a NanoDrop spectrophotometer (Thermo Fisher Scientific). 500 ng of RNA from each sample was reverse transcribed to cDNA using the Thermoscript reverse transcription kit (Invitrogen) with oligo dT primers according to the manufacturer’s instructions. Quantitative PCR was performed on samples in parallel using a MX3000P real time PCR machine (Stratagene, La Jolla, CA) with 1 μl of cDNA (or water control) in a 10 μl final reaction volume using hot-start DNA polymerase (Qiagen, Crawley, United Kingdom) in the presence of SYBR green (Sigma-Aldrich), ROX dye (Invitrogen), and 0.25 μM of specific primers (Table 1), with melt curve analysis of the final product. Data were normalised to endogenous control genes, β-2-microglobulin (*B2M*) and/or β-actin (*ACTB*).

**TABLE 1:** Primer sequences used for quantitative gene expression analysis.

Gene	Accession number	Sense	Antisense	Product size (bp)	Annealing temp. (°C)
*ACTB*	NM_173979.3	AGC AGT CGG TTG GAT CGA GCA	GGG AAG GCA AAG GAC TTC CTG TAA C	137	60
*B2M*	NM_173893.3	GGG TGC TAC ATG TCC ATG TTT GAC C	TGC AGA AGA CAC CCA GAT GTT GAT G	118	60
*ACAN*	NM_173981.2	GAT GCT TCT ATC CCA GCC TCC GC	CGG TCC GGG AAG TGG CGG TAA	125	60
*COL2A1*	NM_001001135.3	ACG TCC AGA TGA CCT TCC TG	GGA TGA GCA GAG CCT TCT TG	126	60
*SIRT1*	NM_001192980.3	CCA GCT AGG ACC ATT ACT GCC	AGC ACA AAC ACA GAT CAT GCA A	145	60
*SIRT3*	NM_001206669.1	CCA CAG ATT AAT GGC GCT GC	GCC CTT CAC ATG GAT CCC AA	145	60

*ACTB*, β-actin; *B2M*, β2 microglobulin; *ACAN*, aggrecan; *COL2A1*, collagen type II, alpha 1; *SIRT1*, Sirtuin 1; *SIRT3*, Sirtuin 3.

### 2.7 Immunofluorescent staining

Parallel cell cultures seeded onto glass coverslips were fixed at indicated time points in 4% paraformaldehyde for 10 min. After washing in sterile phosphate buffered saline, fixed specimens were stored at 4°C until all time points were collected for a given experimental replicate. Coverslips from all time points were processed in the same batch. Cells were permeabilised with 0.5% Triton X–100 in phosphate buffered saline (PBS) for 5 min and blocked with 5% goat serum in 0.1% BSA-PBS (all Sigma–Aldrich) for 1 h. Cells were incubated with primary antibodies in 0.1% BSA-PBS at 4°C overnight as follows: rabbit polyclonal anti-Sirt1 (Abcam ab13749, RRID: AB_300612) at 1:100, mouse monoclonal anti-H3K27me3 (Abcam ab6147, RRID: AB_449502) at 1:500. Following repeated washing in 0.1% BSA-PBS, cells were incubated with goat anti-rabbit Alexa Fluor 555 (Invitrogen A-21430, RRID: AB_2535851) and goat anti-mouse Alexa Fluor 488 (Invitrogen A-11017, RRID: AB_2534084) conjugated secondary antibodies at 1:1000 for 1 h at room temperature. Nuclei were detected with 1 μg/ml 4′,6-diamidino-2-phenylindole (DAPI; Sigma–Aldrich) in PBS. Following washes in PBS, coverslips were mounted with Fluoromount-G (Cambridge Bioscience, Cambridge, United Kingdom).

Immunofluorescently labelled coverslips were imaged on a Leica DMI4000B fluorescent microscope with a 63x/1.30 oil objective and captured as 16-bit images. A custom script implemented using MATLAB (MathWorks, Cambridge, United Kingdom) was used to identify nuclei and analyse nuclear intensity of SIRT1 and H3K27me3. DAPI labelled nuclei were identified using a Canny threshold. The mean intensity within a region of interest specified by each nucleus was evaluated in each fluorescent channel and output for statistical analysis.

### 2.8 Statistical analysis

Statistical analyses were performed using Minitab 18 software (Minitab, Coventry, United Kingdom) or Microsoft Excel. When data sets adhered to a normal distribution, two-way *t*-test, ANOVA, or a general linear model for analysis of variance were used with Bonferroni correction or Tukey tests for multiple comparisons. For non-parametric data sets, Mann–Whitney tests were used to compare conditions. Data quoted in the text are presented as mean ± s.e.m. Details of specific statistical tests and *n* values can be found in the figure legends.

## 3 Results

### 3.1 Physiological glucose and osmolarity enhance *SIRT1* but not *SIRT3* expression

**FIGURE 1:**
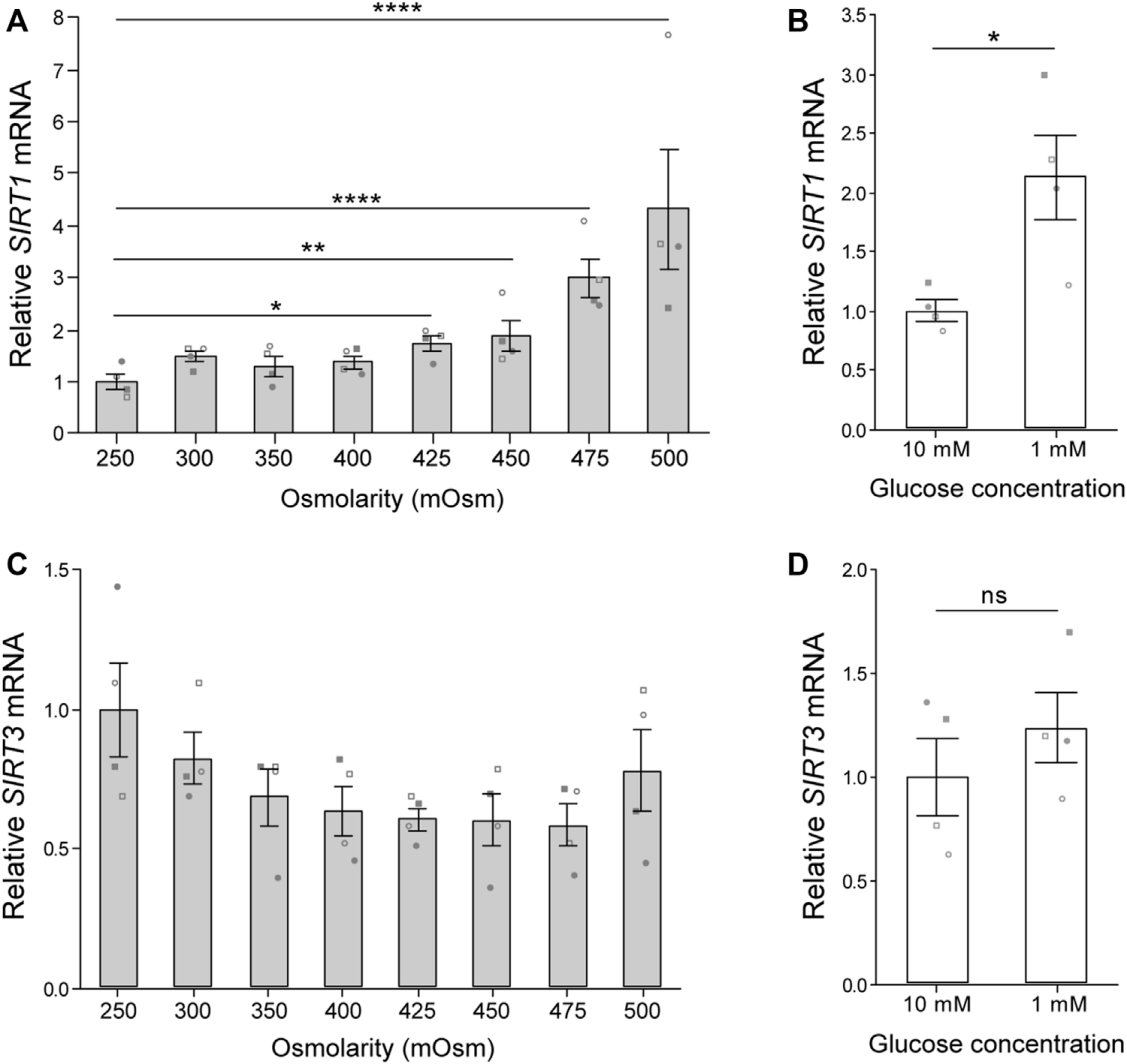
Osmolarity and glucose concentration regulate *SIRT1* but not *SIRT3* gene expression. **(A,B)** Gene expression of **(A)** sirtuin-1 (*SIRT1*) and **(B)** sirtuin-3 (*SIRT3*) normalised to β-2-microglobulin (*B2M*) endogenous control and presented relative to 250 mOsm. Mean ± s.e.m., *n* = 4 donors represented by unique symbols, General linear model with Dunnet multiple comparisons against 250 mOsm: *p = 0.011, **p = 0.005, ****p < 0.0001. **(C,D)** Gene expression of **(C)** *SIRT1* and **(D)** *SIRT3* normalised to β-actin (ACTB) endogenous control and presented relative to 10 mM glucose. Mean ± s.e.m., *n* = 4 donors represented by unique symbols, paired *t*-test: *p = 0.030, n. s. *P* > 0.05. Donors in A,B are distinct from donors in C,D.

The chondrocyte transition from cartilage tissue to monolayer culture is typically accompanied by a shift from a microenvironment with high osmolarity and low glucose to one with low osmolarity and high glucose. Accordingly we assessed the expression of sirtuins 1 and 3 (*SIRT1* and *SIRT3*) in primary chondrocytes subjected to levels of glucose and osmolarity representative of the *in vivo* niche. Exposure of freshly isolated chondrocytes to media with osmolarity ranging from 250 to 500 mOsm for 5 h induced an osmolarity associated increase in *SIRT1* gene expression (Figure 1A). However, *SIRT3* gene expression was not significantly altered (Figure 1B). Culture of chondrocytes in 1 mM glucose for 6 h increased *SIRT1* expression (Figure 1C), but had little effect on *SIRT3* expression (Figure 1D). Accordingly, *SIRT1* is highly responsive to departures from *in vivo* osmolarity and glucose concentrations typically associated with the switch to monolayer culture.

### 3.2 SIRT1 expression declines during *in vitro* monolayer expansion

**FIGURE 2:**
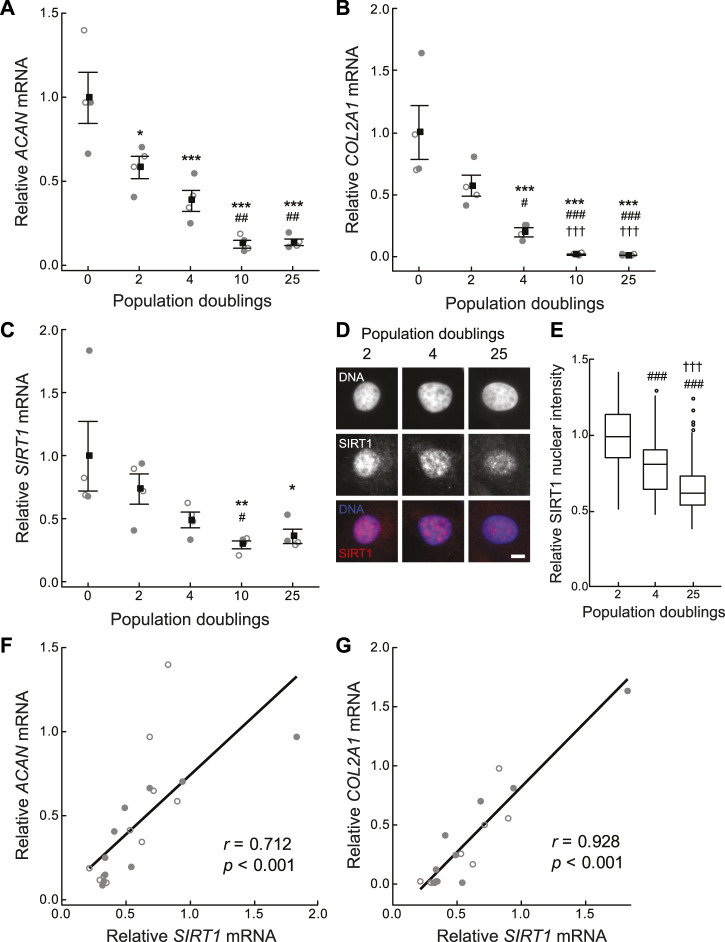
Cartilage matrix and sirtuin 1 gene expression declines during *in vitro* monolayer expansion of primary chondrocytes. **(A–C)** Gene expression of **(A)** aggrecan (*ACAN*), **(B)** collagen type II α-1 chain (*COL2A1*) and **(C)** sirtuin 1 (*SIRT1*) is normalised to β-2-microglobulin (*B2M*) and β-actin (*ACTB*) endogenous control genes and presented relative to 0 population doublings (PDs). Mean ± s.e.m., *n* = 4 from two experiments with individual data points represented by open and closed circles respectively, General linear model with Tukey pairwise comparisons: *p < 0.05, **p < 0.01 and ***p < 0.001 vs. 0 PDs, ^#^ *p* < 0.05, ^##^ *p* < 0.01 and ^###^ *p* < 0.001 vs. 2 PDs, ^†††^ *p* < 0.001 vs. 4 PDs. **(D)** Representative nuclear SIRT1 immunofluorescent staining. Scale bar 5 µm. **(E)** Nuclear SIRT1 protein levels relative to 2 PDs. Boxes represent median and interquartile range, with whiskers extending to 1.5 × interquartile range or the max/min data points, *n*_*2PD*_ = 140, *n*_*4PD*_ = 113, *n*_*25PD*_ = 354 cells, General linear model with Tukey pairwise comparisons: ^###^ *p* < 0.001 vs. 2 PDs, ^†††^ *p* < 0.001 vs. 4 PDs. **(F,G)** Scatter plots of **(F)** *ACAN*, and **(G)** *COL2A1* against *SIRT1* expression. *n* = 20 from two experiments with individual data points represented by open and closed circles respectively, Pearson correlation coefficient (*r*) and *p*-value < 0.001.

Cell therapies typically involve chondrocyte population expansion by monolayer culture *in vitro* before re-implantation into the damaged site at a high cell density. Population expansion is typically limited to 3–4 population doublings (PDs) due to the concomitant loss of phenotypic expression during monolayer culture. In agreement with previous studies (Dell’Accio et al., 2001; Giovannini et al., 2010), a rapid loss of chondrocyte phenotypic marker gene expression for aggrecan (*ACAN*) and collagen type 2 (*COL2A1*) was observed with monolayer expansion (Figures 2A,B). In a new finding, *SIRT1* expression also decreased with increasing PD number, such that a 51.1 ± 6.1% reduction in *SIRT1* mRNA expression occurred by 4 PDs (Figure 2C), with a similar reduction observed for SIRT1 protein expression assessed using immunofluorescent staining (*p* < 0.001; Figures 2D,E). A positive correlation was observed between the loss of *SIRT1* expression and the phenotypic markers *ACAN* (*r* = 0.71, *p* < 0.001) and *COL2A1* (*r* = 0.93, *p* < 0.001); Figures 2F,G). This supports previous findings relating SIRT1 activity and *COL2A1* expression (Oppenheimer et al., 2014).

### 3.3 Chondrocytes have similar morphology and proliferation kinetics in the presence or absence of NAM

To assess whether sirtuin activity during population expansion is required to maintain the regenerative capacity of chondrocytes, medium was supplemented with the sirtuin reaction product and physiological pan-sirtuin inhibitor, nicotinamide (NAM), at 10 mM. NAM binds the regulatory C-pocket of sirtuins to inhibit NAD hydrolysis (Bitterman et al., 2002) and is demonstrated to be capable of abolishing sirtuin enzymatic activity at 10 mM (Guan et al., 2014). In addition to enhancing *SIRT1* gene expression (Figure 1B), glucose deprivation is reported to enhance sirtuin activity by a mechanism linked to the induction of mitochondrial respiration upon glucose scarcity that is hypothesised to increase the NAD/NADH ratio (Lin et al., 2002; Heywood et al., 2006; Heywood et al., 2010). Therefore, chondrocytes expanded under 1 mM glucose were also assessed.

**FIGURE 3:**
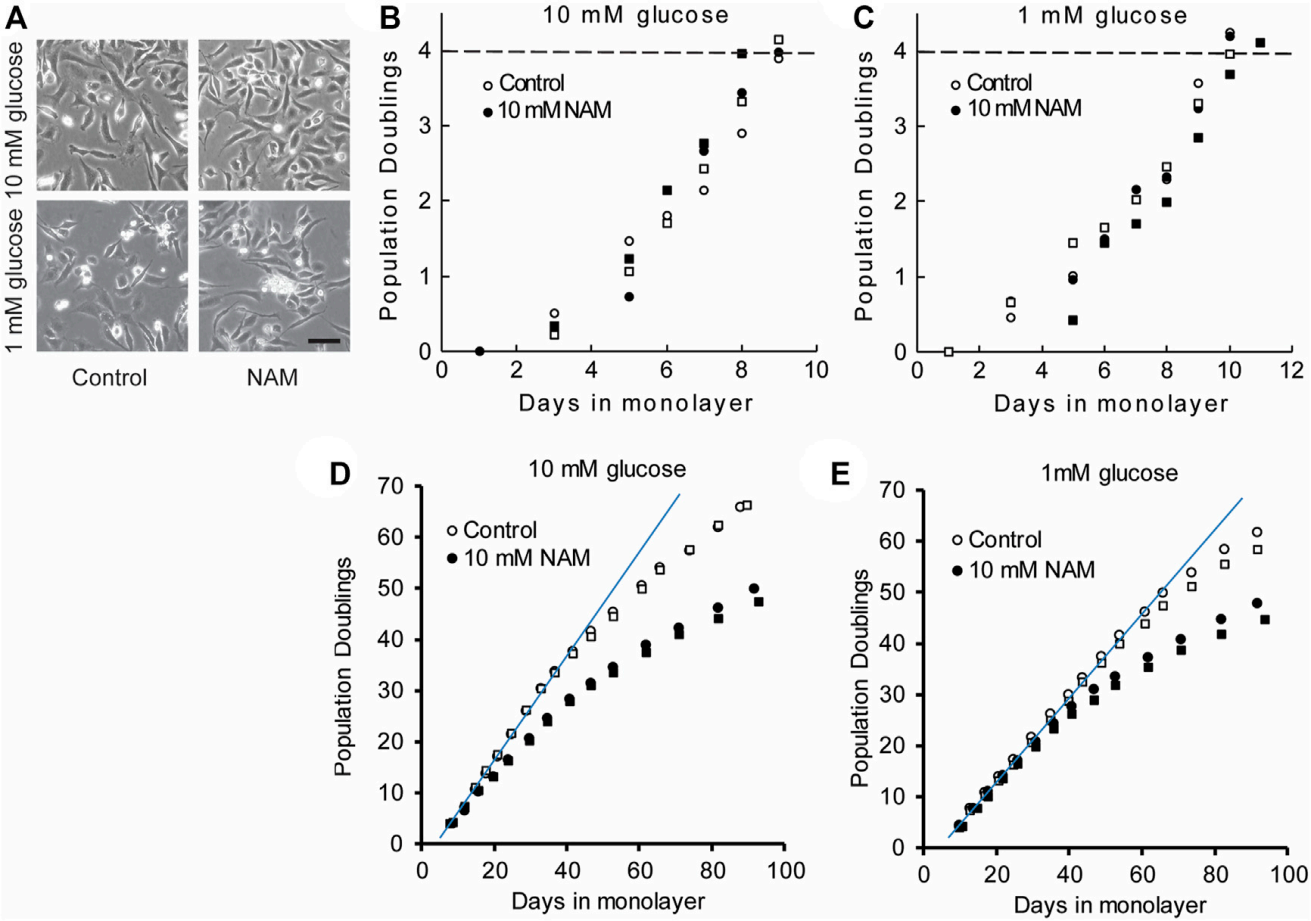
Chondrocytes have similar morphology and early proliferation kinetics in the presence or absence of the sirtuin inhibitor, NAM. **(A)** Chondrocytes expanded in monolayer exhibited similar morphology regardless of glucose concentration or sirtuin inhibition with NAM, illustrated by representative images at day 6. Scale bar 100 µm. **(B,C)** Proliferation kinetics data for chondrocytes expanded for 4PD in **(B)** 10 mM glucose and **(C)** 1 mM glucose with and without NAM. Data from two experiments indicated by circles and squares. The population doubling times calculated from the linear region of the population doubling/days in monolayer plots **(B,C)** were 1.49 ± 0.11 and 1.47 ± 0.12 days (mean ± s.e.m.) for control and NAM treated cells respectively. The final population doubling number at which cells were harvested to prepare cell pellets, indicated by the dashed line in **(B)** and **(C)** was 4.06 ± 0.08 and 4.05 ± 0.06 (mean ± s.e.m.) for control and NAM treated cells respectively. **(D,E)** Long-term proliferation kinetics in which a linear fit indicates the trajectory of the initial linear growth phase. During long-term culture, premature proliferative arrest was observed in the presence of NAM treatment for both 10 mM and 1 mM glucose media **(D,E)**, illustrated by earlier deviation from linear growth compared to cells without NAM.

Chondrocytes expanded in monolayer exhibited similar morphology regardless of glucose concentration or sirtuin inhibition with NAM (Figure 3A). Population doubling time (PDT) was calculated from the linear proliferation phase up to 4 PDs. NAM did not markedly alter PDT at early timepoints, which was 1.33 and 1.37 days in media containing 10 mM glucose in the presence and absence of NAM, respectively (Figure 3B). Culture under 1 mM glucose conditions slightly increased these respective PDT to 1.65 and 1.57 days in the presence and absence of NAM, respectively (Figure 3C). Thus, expansion in 1 mM glucose resulted in a delay of 1–2 days in reaching four population doublings when compared to 10 mM glucose. Longer term, cells cultured in the presence of NAM treatment initiated proliferative arrest earlier than in the absence of NAM, illustrated by the premature deviation from the linear initial proliferation rate (Figures 3D,E) and consistent with the known role of SIRT1 antagonising senescence (Bitterman et al., 2002; Li and Tollefsbol, 2011).

### 3.4 Treatment with the sirtuin inhibitor, NAM, during monolayer expansion diminished chondrocyte regenerative capacity

**FIGURE 4:**
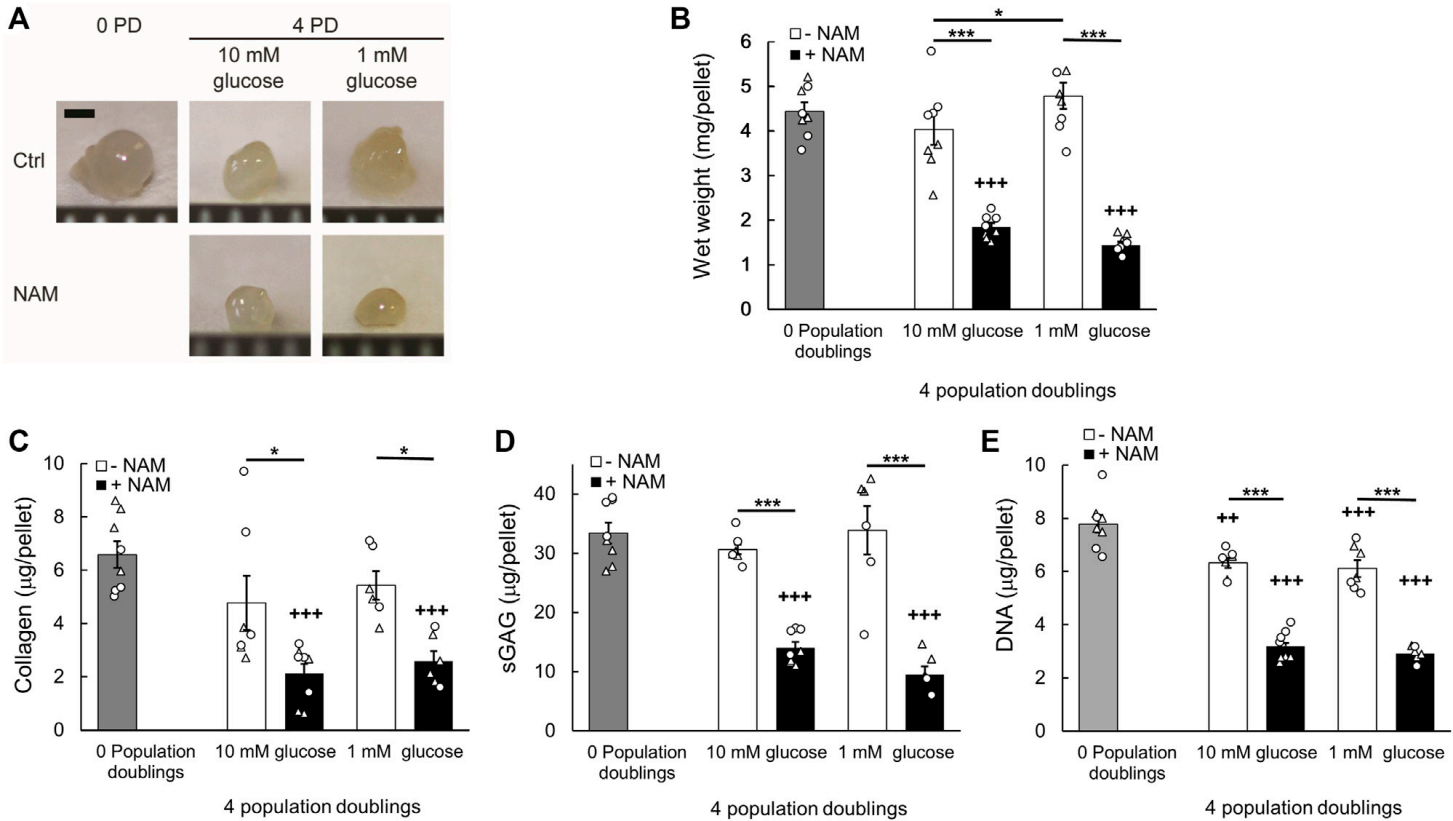
Inhibition of sirtuin activity during monolayer expansion abrogates chondrocyte regenerative capacity. **(A)** Representative cell pellets at day 28 of 3D culture created from chondrocytes which were either freshly isolated, 0 population doublings (0 PD), or monolayer expanded for 4 PDs in media containing either 10 mM or 1 mM glucose in the presence or absence of the sirtuin inhibitor nicotinamide (NAM). Scale bar 1 mm. **(B)** Pellet wet weight, **(C)** collagen content, **(D)** sulphated glycosaminoglycan (sGAG) content, and **(E)** DNA content at day 28. Mean ± s.e.m, *n*_*0PD*_ = 8 **(B–E)**, *n*_*4PD 10mM Ctrl*_ = 9 **(B)** 7 **(C–E)**, *n*_*4PD 10mM NAM*_ = 10 **(B)** 8 **(C–E)**, *n*_*4PD 1mM Ctrl*_ = 10 **(B)** 6**(C–E)**, *n*_*4PD 1mM NAM*_ = 12 **(B)** 6 **(C–D)** 7 **(E)** from two experiments indicated by symbol shape, General linear model with Tukey pairwise comparisons: *p < 0.05, ***p < 0.001; ^+++^ *p* < 0.001 vs. 0 PD.

3D pellet cultures recapitulate the re-implantation phase of cell therapy techniques such as ACI, where the cells must be able to regenerate a cartilaginous matrix for clinical success. Cells were recovered from monolayer following 4 PDs and 3D pellets were prepared (Giovannini et al., 2010). The pellets were cultured for a further 28 days, using control media for all groups. The equivalence in population doubling prior to cell pellet formation, illustrated in Figures 3B,C, is important because this is linked to regenerative capacity (Dell’Accio et al., 2001; Giovannini et al., 2010). Cells cultured in monolayer for 4 PDs in the presence of the sirtuin inhibitor, NAM, generated smaller tissue mass after 28 days pellet culture compared with both the untreated control cells and freshly isolated cells that had not been expanded in monolayer prior to pellet formation (0 PD; Figures 4A,B). Wet weight was significantly reduced with expansion in NAM to 44.17 ± 0.02% (*p* < 0.001) of that of untreated controls for 10 mM glucose cultures (Figure 4B). Tissue composition mirrored wet weight, with significantly reduced collagen (41.51 ± 0.08%, *p* < 0.05) and sGAG (45.78 ± 0.03%, *p* < 0.001) content in pellets from cells exposed to NAM treatment during the preceding expansion phase (Figures 4C,D). Glucose deprivation is reported to increase sirtuin activity (Kaeberlein et al., 2002; Lin et al., 2002) and expression (Heywood et al., 2016). Expansion in media containing 1 mM glucose resulted in cells that produced significantly greater tissue mass upon 3D pellet culture compared to cells expanded in media containing 10 mM glucose (*p* < 0.05; Figure 4B). This effect was abolished by the presence of NAM during expansion, providing a significant interaction between glucose concentration and NAM supplementation (*p* = 0.002; Figure 4B). Trends in pellet DNA content at day 28 mirrored ECM, with freshly isolated (0 PD) and control cells proliferating during the 3D pellet phase, with a 58%–102% increase in DNA (Figure 4E). By contrast, NAM treated chondrocytes which proliferated as normal in 2D (Figures 3B,C) failed to demonstrate an increase in DNA on incorporation into 3D pellet culture (Figure 4E). Accordingly, inhibition of sirtuin activity during the monolayer expansion phase is linked to a marked reduction in cartilage tissue regenerated upon transfer to 3D pellet culture.

### 3.5 SIRT1 expression correlates with epigenetic chromatin modification in monolayer

**FIGURE 5:**
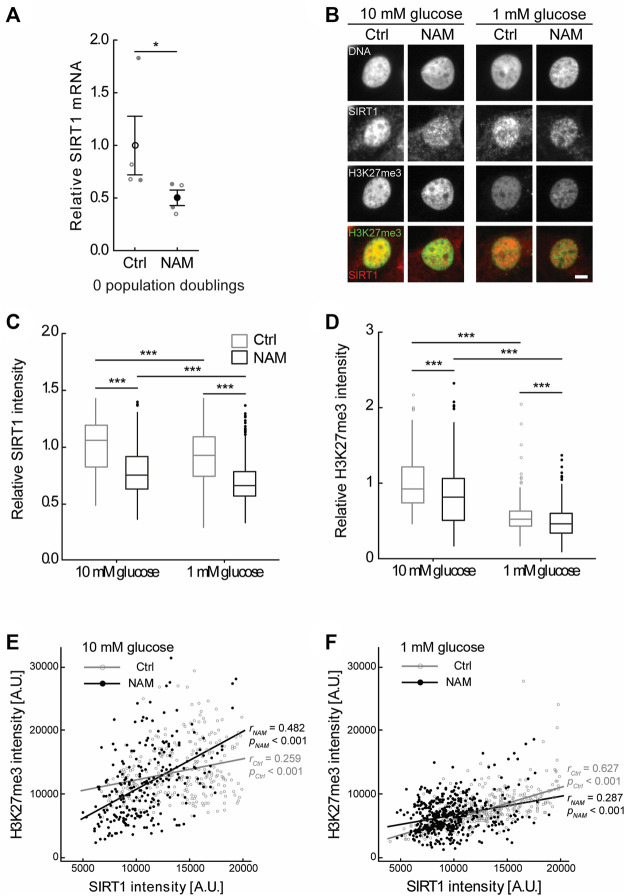
Treatment with the sirtuin inhibitor, NAM, decreased expression of SIRT1 and abundance of the epigenetic mark H3K27me3. **(A)** Treatment of primary (0 PD) chondrocytes for 24 h with 10 mM NAM significantly inhibited *SIRT1* gene expression. Mean ± s.e.m., *n* = 4 from two experiments with individual data points represented by open and closed circles respectively, Mann-Whitney test: *p < 0.05. **(B)** Representative SIRT1 and H3K27me3 immunofluorescent nuclear staining in chondrocytes expanded for four population doublings (PDs) in media containing either 10 mM or 1 mM glucose in the presence or absence of the sirtuin inhibitor nicotinamide (NAM). Scale bar 5 µm. **(C)** Nuclear SIRT1 and **(D)** H3K27me3 levels in chondrocytes at 4 PDs relative to 10 mM glucose control cells. Boxes represent median and interquartile range, with whiskers extending to 1.5 × interquartile range or the max/min data points. **(E,F)** Scatter plots of nuclear H3K27me3 against nuclear SIRT1 intensity in 4 PD chondrocytes cultured in **(E)** 10 mM glucose or **(F)** 1 mM glucose in the presence or absence of NAM. *n*_*10mM Ctrl*_ = 270 cells, *n*_*10mM NAM*_ = 246 cells, *n*_*1mM Ctrl*_ = 416 cells, *n*_*1mM NAM*_ = 468 cells from two experiments. **(C,D)** Mann-Whitney test: ***p < 0.001. **(E,F)** Pearson correlation coefficient (r) and *p*-value presented.

Treatment with the sirtuin inhibitor NAM, restricted to the monolayer expansion phase, markedly altered regeneration in the subsequent 3D pellet (Figure 4). However, there was no evidence that the presence of NAM significantly affected the time course of *ACAN* and *COL2A1* gene expression over the preceding 4 PDs in monolayer (Supplementary Figures S2A,B). Therefore, we examined evidence of epigenetic changes, consistent with an enduring cell phenotype. The chromatin mark H3K27me3 is associated with heterochromatin formation and gene silencing and has an important role in development and differentiation (Kuzmichev et al., 2002; Bosch-Presegue and Vaquero, 2015; Lui et al., 2016). Furthermore, SIRT1 is reported to participate in the polycomb repressive complex 4 (PRC4), a complex with H3K27me3 histone methyltransferase activity (Bosch-Presegue and Vaquero, 2015). In this study, H3K27me3 immunofluorescent intensity correlated positively with SIRT1 expression in the nuclei of chondrocytes cultured in both 10 mM and 1 mM glucose to 4 PDs (Figure 5). Treatment with the sirtuin inhibitor, NAM, during monolayer expansion decreased nuclear levels of both SIRT1 and H3K27me3 (Figures 5A–D) and weakened the correlation between these (Figures 5E,F). These data support the proposal that sirtuins are involved in the maintenance of this chromatin mark during monolayer expansion.

### 3.6 Pharmacological augmentation of sirtuin activity in monolayer enhances pellet culture mass

**FIGURE 6:**
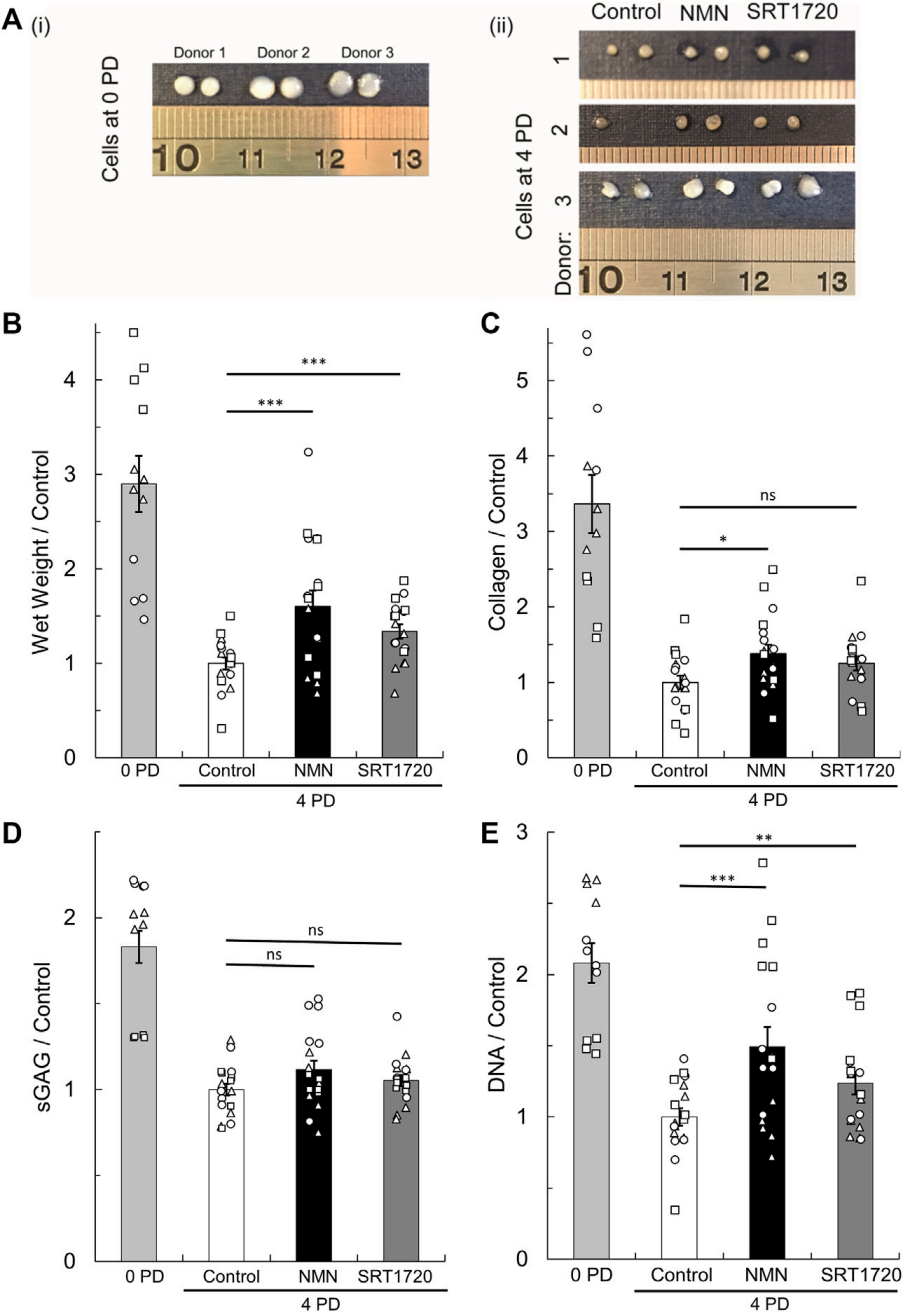
Regeneration of cartilage tissue following transfer of monolayer cells to 3D pellet culture. **(A)** Representative tissue masses formed after 21 days culture of cell pellets created from (i) freshly isolated cells (0 PD) or (ii) monolayer expanded for four population doublings (4 PDs) in media containing 10 mM glucose supplemented with the pan-sirtuin activator, 100 µM NMN, or the SIRT1 specific activator, 100 nM SRT1720, or untreated controls. Scale units cm. **(B)** Pellet wet weight, **(C)** collagen content, **(D)** sulphated glycosaminoglycan (sGAG) content, and **(E)** DNA content at day 21 relative to the 4 PD control. Mean ± s.e.m, *n*_*0PD*_ = 12, *n*_*4PD*_ = 18 from three donors indicated by symbol shape. 2-factor ANOVA with replication and bonferroni correction: *p < 0.05, **p < 0.01 ***p < 0.001, ^ns^ *p* > 0.05. The cell number seeded to pellets for each treatment group is shown in Supplementary Figure S3.

Next, we tested whether augmentation of sirtuin activity in chondrocytes during monolayer culture enhances subsequent cartilage tissue generation upon transfer to a cell pellet culture model of re-implantation. 100 μM NMN or 100 nM SRT1720 (Milne et al., 2007), were added during chondrocyte expansion to 4 PDs, at which point 3D pellets were prepared. NMN is anticipated to activate the whole sirtuin family (SIRT1-7) by acting as the precursor of their common sirtuin substrate, NAD (Imai, 2010; Imai and Guarente, 2014), whereas the synthetic sirtuin activator SRT1720 has specificity for SIRT1 (Milne et al., 2007). During expansion, chondrocytes treated with NMN and SRT1720 had similar proliferation rates compared to untreated controls (data not shown). Day 21 pellets are illustrated in Figure 6A and compared to cell pellets which were prepared from the freshly isolated cells (0 PD) to indicate the performance of fully differentiated cells from each donor. The wet weights were significantly greater for cells expanded in the presence of sirtuin activators, being 60% and 34% greater compared to untreated controls, for NMN and SRT1720 respectively (*p* < 0.001; Figure 6B). Analysis of the tissue composition (Figures 6C–E) indicated that the key effect was improved maintenance of cell number during the pellet phase. In untreated cells, measured cell numbers were lower after pellet culture than when seeded within the pellet, suggesting loss of viability (Figure 6E). Additionally, the tissue regenerated by the NMN treatment group had 38% greater collagen content (*p* < 0.05) while sGAG levels were not significantly enhanced.

### 3.7 The beneficial effects of sirtuin activation are culture context dependent

**FIGURE 7:**
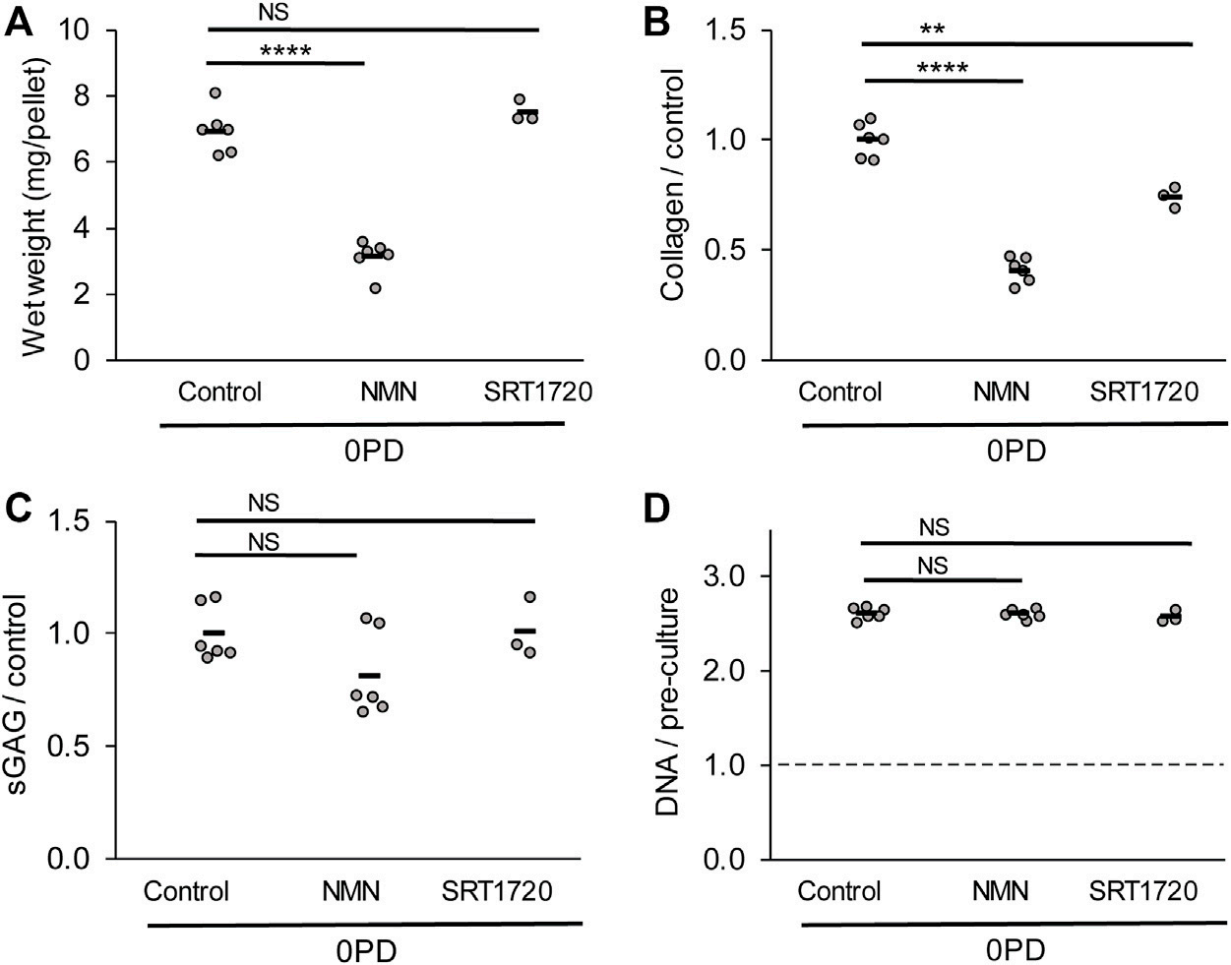
Regeneration of cartilage tissue by freshly isolated (0 PD) chondrocytes is impaired by the presence of sirtuin activators during 3D pellet culture. **(A)** Cell pellet wet weights, **(B)** collagen, **(C)** sulphated glycosaminoglycan and **(D)** DNA content after 21 days pellet culture in the presence or absence of sirtuin activators 100 µM NMN or 100 nM SRT1720. DNA content is normalised to pre-culture pellet values at day 0. *n* = 6 for all groups except SRT1720 where *n* = 3. The freshly isolated (0 PD) cells were pooled from four donors before pellet formation. Two-way *t*-test with Bonferroni correction: ***p < 0.001 ****p < 0.0001, ^NS^ *p* > 0.05.

Freshly isolated chondrocytes (0 PD) were prepared as cell pellets without prior monolayer culture, to examine the effect of sirtuin activation during the 3D culture phase (Figure 7). Unexpectedly, the presence of NMN during 3D pellet culture resulted in a 55% reduction in the tissue mass compared to untreated controls (*p* < 0.0001; Figure 7A). Both NMN and SRT1720 reduced collagen content, by 59% (*p* < 0.0001) and 25% (*p* < 0.01) respectively, while sGAG and DNA content were unaffected. Therefore, treatment of differentiated cells with sirtuin activators in a 3D tissue formation context may be detrimental to tissue regeneration, with implications for approaches that aim to use delivery of activators to enhance repair *in situ*.

## 4 Discussion

In this study, we examined the potential role of small molecule modulators of sirtuins to augment the regenerative potential of articular chondrocytes, using methods that replicate those adopted for the expansion of cells in regenerative therapies for cartilage repair. This study has demonstrated that *SIRT1* gene expression progressively declines with culture duration during *in vitro* monolayer expansion, and that pharmacological modulators of sirtuin activity during *in vitro* monolayer culture influence the ability of expanded chondrocytes to regenerate a cartilaginous matrix upon transfer to a 3D culture environment. Importantly the regenerative capacity of monolayer-expanded chondrocytes can be manipulated *via* activation or inhibition of sirtuin activity during the expansion phase alone. We observed that SIRT1 gene expression diminishes during monolayer culture (Figure 2). Moreover, inhibition of sirtuin activity during the expansion phase, using NAM, further reduced regenerative capacity during subsequent 3D culture. Accordingly, we tested whether media supplementation during monolayer expansion with known activators of sirtuins would enhance the ability of the cells to synthesise a cartilaginous ECM in 3D, as a novel means of enhancing regenerative medicine approaches to cartilage repair.

Nicotinamide mononucleotide (NMN) is a precursor of the common sirtuin substrate, nicotinamide adenine dinucleotide (NAD), and has been demonstrated by others to enhance sirtuin activity when supplemented in the diet, or in culture medium of cells *in vitro* (Imai, 2010). Its efficacy in enhancing chondrocyte NAD levels *in vitro* was confirmed in our hands (Supplementary Figure S1). In addition, we examined the effect of the synthetic SIRT1-specific activator, SRT1720 (Milne et al., 2007). Cells expanded in the presence of either sirtuin activator generated significantly greater cartilage tissue mass on transfer to 3D culture (Figure 6). This was attributed both to an increase in cell number in the mature 3D tissue (NMN and SRT1720 treatments) and an increased accumulation of collagen ECM (NMN treatment) compared to controls. The positive regulation of cartilage-specific matrix gene expression, such as type II collagen, is an established role of SIRT1 in cartilage (Dvir-Ginzberg et al., 2008). However, comparison of the regenerated tissue composition, particularly cellularity (Figure 6E), suggests that the predominant effect of sirtuin activation in this system is to support the survival and proliferation of culture expanded chondrocytes following transfer from monolayer to a 3D environment. Consistent with this, expansion in the presence of the sirtuin inhibitor, NAM, produced the opposite effect, with significantly reduced cell numbers in the regenerated tissues (Figure 4E). Based on 7.7 pg of DNA per chondrocyte (Kim et al., 1988), we observe an approximate two-fold increase in cellularity in the 0 PD and control 4 PD conditions while overall cell number remains unchanged with NAM treatment (Figure 4E). The relative contribution of proliferation and apoptosis to these observations could be explored in the future.

These data are consistent with the pro-survival actions of sirtuins, which are reported to block apoptotic cell death under stress conditions, through a variety of mechanisms including inhibition of the pro-apoptotic protein, p53 (Luo et al., 2001; Vaziri et al., 2001; Takayama et al., 2009; Gagarina et al., 2010), Ku70 (Cohen et al., 2004), forkhead transcription factors (Motta et al., 2004), and p65 (Yeung et al., 2004). Alternatively, sirtuins may promote cell survival through crosstalk with hypoxia-inducible factor 1-α (HIF1α). It is reported that SIRT1 depletion or inactivation hyperacetylates HIF1α and reduces HIF1α accumulation under hypoxic conditions. Chondrocytes become increasingly dependent on oxidative phosphorylation within the aerobic environment in monolayer culture, which contrasts with the highly glycolytic metabolism of primary cells (Heywood and Lee, 2010, Heywood and Lee, 2017). The activation of sirtuins may therefore promote cell survival on transfer to 3D pellet culture *via* HIF1α activation, which adapts cell metabolism to the limited oxygen within the pellet culture. SIRT1 is established to modulate HIF1α activity and expression *via* direct interaction (Laemmle et al., 2012; Joo et al., 2015) or epigenetic modification of the HIF1α promoter (Dong et al., 2016; Luo and Wang, 2018). Further work is required to determine which mechanisms mediate the enhanced cellularity of tissues following cell expansion with sirtuin activators observed in this study.

NAM was used in this study as it is reported to bind to the regulatory C-pocket of sirtuins and inhibit their enzymatic activity (Bitterman et al., 2002; Guan et al., 2014). The supplementation or depletion of NAM is reported to inhibit or activate sirtuins, respectively (Luo et al., 2001; Anderson et al., 2003; Gallo et al., 2004). However, some NAM may be converted into NAD *via* the NAD salvage pathway, the rate limiting step of which is the conversion of NAM to NMN by nicotinamide phosphoribosyl transferase (NAMPT). Therefore, NAM treatment has the potential to indirectly activate sirtuins. However, the present study observed that chondrocytes treated with the putative sirtuin inhibitor NAM had the opposite response to treatment with the NAD precursor, NMN (compare Figures 4, 6). Therefore, the inhibitory effects of NAM treatment appear to outweigh any effects from *de-novo* NAD synthesis in this study. Differences in the efficacy of NAM as a sirtuin inhibitor may occur due to differential expression of NAMPT. For example, normal chondrocytes such as those used in this study are reported to express low levels of NAMPT compared to cells derived from OA tissue used in other studies (Dvir-Ginzberg et al., 2008). Indeed, NAMPT has also been shown to be upregulated in interleukin-1β mediated chondrocyte dedifferentiation, leading to SIRT1 activation (Hong et al., 2011). Further support for NAM as an inhibitor of sirtuins is shown in the long-term proliferation data (Figures 3D,E). As discussed above, sirtuins are established to antagonise cell apoptosis and growth arrest and we observed that the presence of NAM during long-term monolayer culture resulted in premature onset of proliferative growth arrest.

Sirtuins are involved in epigenetic regulation of numerous genes. For example, established SIRT1 targets include histones such as histone H4 lysine 16 (H4K16), histone H3K9, and H3K14 which regulate chromatin structure and biological function (Imai et al., 2000; Bosch-Presegue and Vaquero, 2015). Sirtuins are also involved in histone methylation, by modulating the activity of several methyltransferases (Roth and Chen, 2014; Bosch-Presegue and Vaquero, 2015). For example, SIRT1 associates with enhancer of zeste homolog 2 (EZH2) within polycomb repressive complexes (Kuzmichev et al., 2005), an enzyme with H3K27me3 histone methyltransferase activity (Bosch-Presegue and Vaquero, 2015). H3K27me3 is associated with heterochromatin formation and gene silencing and has an important role in development, differentiation and cell memory (Margueron and Reinberg, 2011; Bosch-Presegue and Vaquero, 2015; Heo et al., 2015; Marasca et al., 2018). H3K27 methylation plays a critical role in chondrocyte proliferation and hypertrophy in the growth plate (Lui et al., 2016), while inhibition of EZH2 histone-lysine N-methyltransferase activity, that leads to reduced H3K27me3, ameliorates OA development (Chen et al., 2016). Consistent with other studies, this study identified a correlation between SIRT1 expression and H3K27me3 (Vazquez et al., 2018) and found that the sirtuin inhibitor NAM reduced H3K27me3 (Figures 5B,D). This is consistent with a role of sirtuins in the maintenance of this chromatin mark during monolayer expansion of chondrocytes. Accordingly, the pharmacological modulation of sirtuin activity during monolayer expansion may leave an epigenetic fingerprint on the cells which could convey persistent effects on cell function following re-implantation.

Such persistence post-expansion may be advantageous as effects gained by *in vitro* treatment with sirtuin activators can be maintained following wash-out of the drug prior to transplantation, thereby avoiding direct exposure of the patient to the sirtuin modulating drugs. Previous reports have suggested that overexpression of sirtuins *in vivo* may have detrimental effects, for example SIRT1 and SIRT7 overexpression is reported to be an indicator of poor prognosis in some cancers (Deng et al., 2018; Ma et al., 2018). Moreover, in this study we observed that primary chondrocytes treated directly in pellet culture with sirtuin activators behaved differently from monolayer-expanded cells with markedly decreased matrix synthesis (Figure 7). Together these finding suggest that restricting modulation of sirtuin activation to the *in vitro* expansion phase of cellular therapies may deliver a number of benefits.

NMN is a common activator for all sirtuins by acting as a precursor for their substrate NAD (Imai, 2010; Imai and Guarente, 2014). By contrast, the synthetic sirtuin activator, SRT1720, has many hundred-fold greater potency for SIRT1 than the closest homologues (Milne et al., 2007). The similar response to each activator illustrated in Figure 6 supports the hypothesis that the effect of sirtuin activation on cartilage regeneration may be attributable, at least in part, to SIRT1. Unexpectedly, this study found that the application of the pan-sirtuin activator, NMN, to freshly isolated chondrocytes in a 3D pellet inhibited tissue regeneration (Figure 7), particularly decreasing wet weight and collagen content. The mechanism is unknown and could be the subject of future studies. One potential cause may be due to the co-activation of SIRT7. In direct contrast to SIRT1 and SIRT6 which are required for cartilage homeostasis (Nagai et al., 2015; Wu et al., 2015; Fu et al., 2016), the selective knockdown of SIRT7 was reported to proportionally increase matrix synthesis in chondrocytes (Korogi et al., 2018). This highlights the importance of specificity of selected activators to sirtuin family members. Smith et al. (2022) recently used pellet culture to induce chondrogenic differentiation in human embryonic stem cells and found that activation of SIRT1 using 5 μM SRT1720 during pellet culture leads to increased collagen type II and aggrecan gene and protein expression, but reduced sGAG accumulation. This contrasts with our findings with mature freshly isolated bovine chondrocytes where we observed a decrease in total collagen with a 50 × lower dose of SIRT1720 (Figure 7B), indicating that the response to SIRT1 activation may depend on dose and the differentiation state of the chondroprogenitor cell or chondrocyte. These studies highlight the importance of the context in which sirtuin activators are applied. For example, although sirtuin activators may be beneficial if applied in monolayer or during differentiation, further research is required to determine whether they may be detrimental to healthy cartilage if given systemically to a patient or injected into the joint.

The use of sirtuin activators during the cell processing stage has potential to enhance the outcome of cell-based cartilage repair techniques such as ACI. However, the regenerative capacity of expanded chondrocytes was not restored to that of freshly isolated (0 PD) cells. Further dose-optimisation may be achieved, but the efficacy of small molecule sirtuin activators will be dependent on the retained expression of the target sirtuins, which we have demonstrated to decline *in vitro* (Figures 2C–D). Similarly, the expression of SIRT1 is reported to be reduced in chondrocytes derived from OA tissue (Dvir-Ginzberg et al., 2008). Thus, variations in sirtuin expression may explain the variable response of cells isolated from different donors to sirtuin activators following equivalent population expansion (Figure 6), in addition to other biological variation. Therefore, research effort should be directed toward identifying the physiological conditions that support the retention of sirtuin expression *in vitro*.

The present study demonstrates for the first time that modulation of sirtuin activity during *in vitro* monolayer expansion influences subsequent chondrocyte regenerative potential. Given that *in vitro* monolayer expansion is a requirement of cell therapy-based cartilage repair strategies, these findings have relevance to the clinical success of cartilage repair procedures. Significant increases in regenerated tissue mass, cell survival and collagen content were achieved by supplementing expansion media with selected small molecule sirtuin activators. Importantly, however, the use of these sirtuin activators on differentiated cells *in situ* may be contraindicated.

## Data Availability

The original contributions presented in the study are included in the article/Supplementary Materials, further inquiries can be directed to the corresponding author.
