# Supplementary material for: Modulation of sirtuins during monolayer chondrocyte culture influences cartilage regeneration upon transfer to a 3D culture environment
Source: Front Bioeng Biotechnol. 2022 Dec 6;10:971932. doi: 10.3389/fbioe.2022.971932 (PMC9763269; doi:10.3389/fbioe.2022.971932)
Supplement: Supplementary file 1 [file DataSheet1.DOCX]

Supplementary Material

# Supplementary Figures


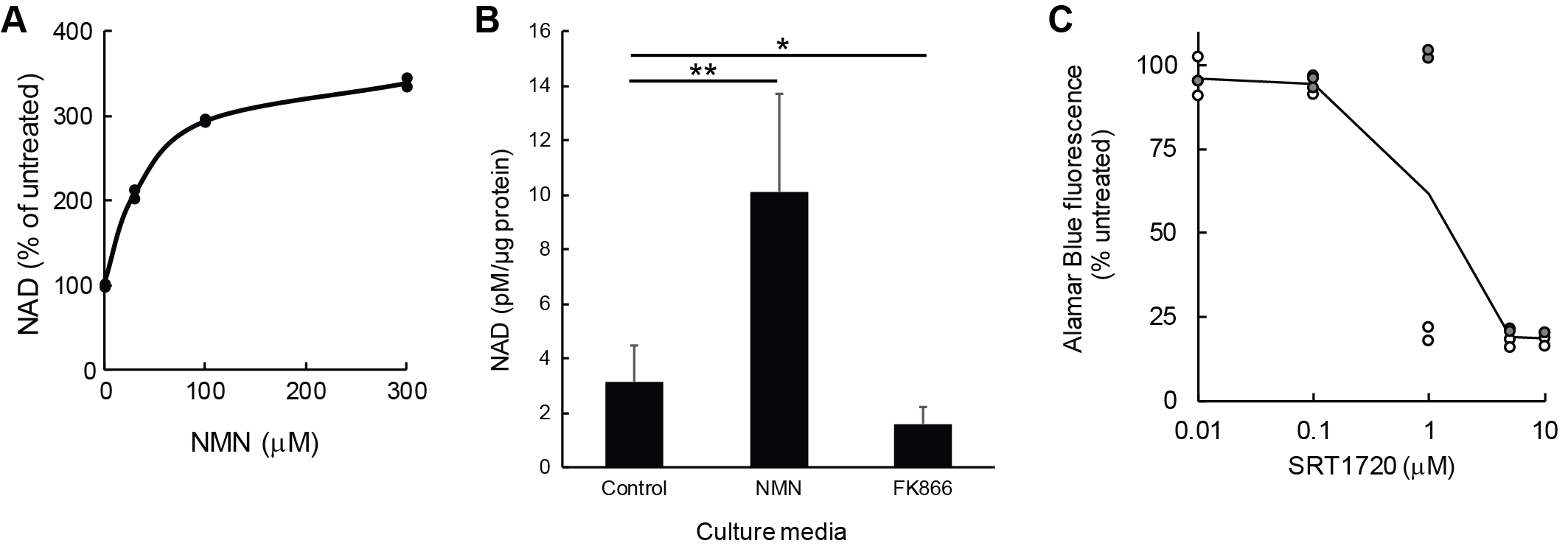


**Supplementary Figure S1.** Nicotinamide mononucleotide (NMN) increases chondrocyte levels of the sirtuin substrate, nicotinamide adenine dinucleotide (NAD), in a dose-dependent manner. **(A)** Duplicate data points for each dose of NMN from 0-300 μM are shown. An effective concentration of 100 μM NMN was selected from for further experiments. **(B)** 100 μM NMN significantly increased NAD levels, whereas the negative control, 10 nM FK866, decreased these values as expected. Mean ± s.d., *n* = 5, ANOVA: **p* < 0.05, ***p* < 0.01. **(C)** A concentration of 100 nM SRT1720 was selected from the effective range established by Milne et al. (2007) and was confirmed to be compatible with maintenance of chondrocyte viability and metabolic function over many days required for cell expansion by Alamar blue assay, and cell morphology (not shown). Mean ± s.e.m., *n* = 4 from 2 donors.


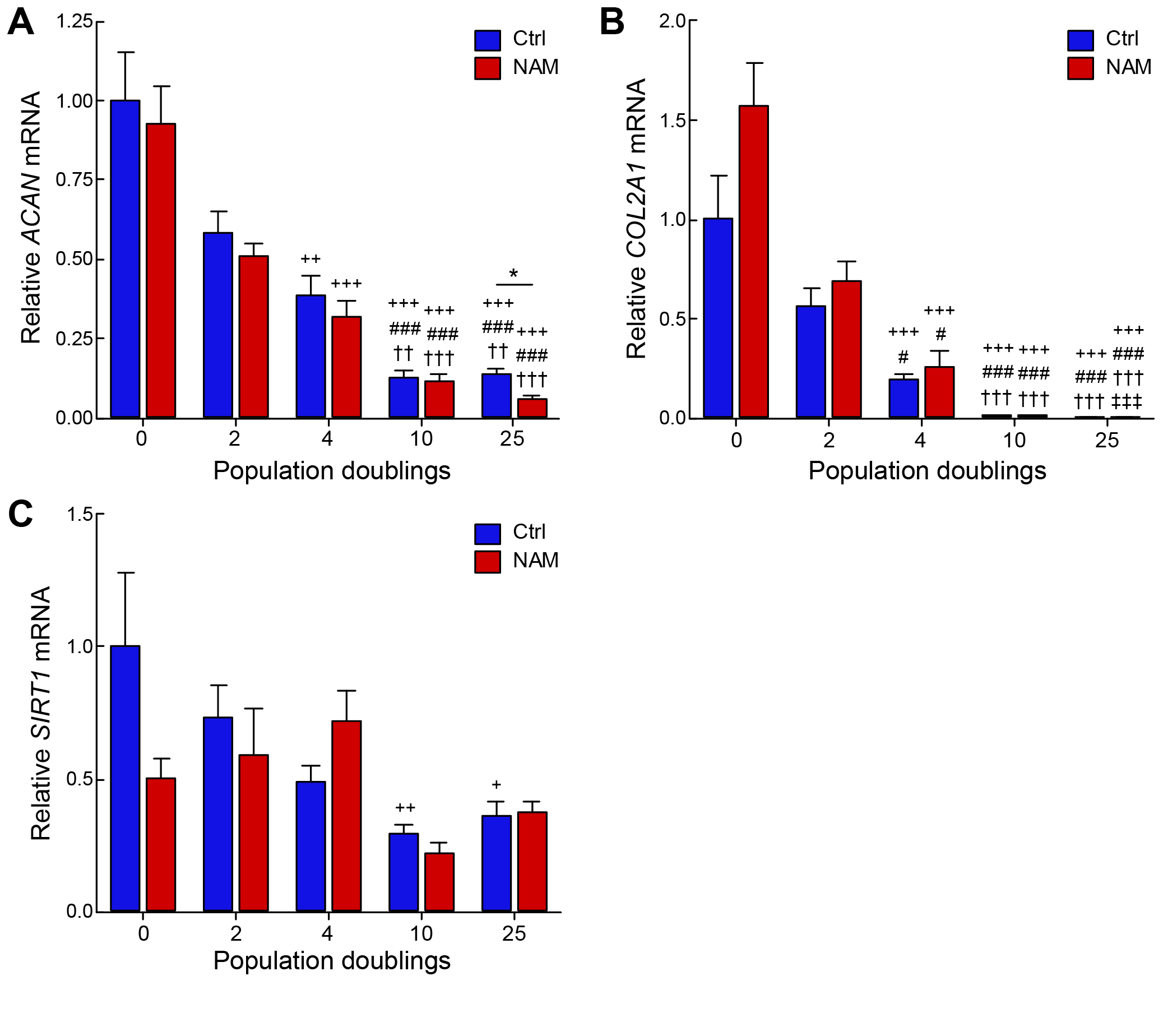


**Supplementary Figure S2.** Treatment of primary chondrocytes with nicotinamide (NAM) does not alter population doubling dependent expression of aggrecan or collagen type 2, but disrupts expression of Sirtuin 1. **(A)** Aggrecan (*ACAN*) gene expression. **(B)** Collagen type II α1 chain (*COL2A1*) gene expression. **(C)** Sirtuin 1 (*SIRT1*) gene expression. Gene expression normalised to the geometric mean of beta-2 microglobulin (*B2M*) and β-actin (*ACTB*) endogenous control genes, and expressed relative to 0 population doublings (PDs) control (Ctrl). Mean ± s.e.m, *n_2PD NAM_* = 3, *n_all other groups_* = 4 from 2 experiments, General linear model with Tukey pairwise comparisons: **p* < 0.05; ^++^*p* < 0.01 and ^+++^*p* < 0.001 vs. 0 PDs, ^#^*p* < 0.05 and ^###^*p* < 0.001 vs. 2 PDs; ^††^*p* < 0.01 and ^†††^*p* < 0.001 vs. 4 PDs, ^‡‡‡^*p* < 0.001 vs. 10 PDs.

**Supplementary Figure S3.** The DNA content of a sample of cell pellets harvested immediately after pellet formation. DNA content is illustrated as millions of cells/pellet using an estimated 7.7 pg DNA per cell. Mean and s.e.m. of 4 pellets per treatment group. Immediately prior to pellet formation, cells were monolayer expanded for 4 population doublings (4 PDs) in media containing 10 mM glucose supplemented with the pan-sirtuin activator, 100 µM NMN, or the SIRT1 specific activator, 100 nM SRT1720, or untreated controls.

# References

Milne, J.C., Lambert, P.D., Schenk, S., Carney, D.P., Smith, J.J., Gagne, D.J., et al. (2007). Small molecule activators of SIRT1 as therapeutics for the treatment of type 2 diabetes. *Nature* 450(7170)**,** 712-716. doi: 10.1038/nature06261.
